# Natural product triptolide induces GSDME-mediated pyroptosis in head and neck cancer through suppressing mitochondrial hexokinase-ΙΙ

**DOI:** 10.1186/s13046-021-01995-7

**Published:** 2021-06-09

**Authors:** Jing Cai, Mei Yi, Yixin Tan, Xiaoling Li, Guiyuan Li, Zhaoyang Zeng, Wei Xiong, Bo Xiang

**Affiliations:** 1 Hunan Provincial Cancer Hospital and Cancer Hospital Affiliated to Xiangya Medical School, Central South University, Tongzipo Road, Changsha, 410013 Hunan China; 2 Hunan Key Laboratory of Nonresolving Inflammation and Cancer, The Third Xiangya Hospital, Central South University, Changsha, 410013 Hunan China; 3 The Key Laboratory of Carcinogenesis of the Chinese Ministry of Health, Xiangya Hospital, Central South University, Changsha, 410008 Hunan China; 4 The Key Laboratory of Carcinogenesis and Cancer Invasion of the Chinese Ministry of Education, Cancer Research Institute and School of Basic Medical Sciences, Central South University, Changsha, 410078 Hunan China; 5 Department of Dermatology, Xiangya Hospital, Central South University, Changsha, 410008 Hunan China; 6 Department of Dermatology, Second Xiangya Hospital, Hunan Key Laboratory of Medical Epigenetics, The Central South University, Changsha, 410011 Hunan China

**Keywords:** Head and neck squamous cell carcinoma, Ferroptosis, Gasdermins, Nasopharyngeal carcinoma, X(c)(−) cysteine/glutamate antiporter

## Abstract

**Background:**

Pyroptosis is a lytic cell death form executed by gasdermins family proteins. Induction of tumor pyroptosis promotes anti-tumor immunity and is a potential cancer treatment strategy. Triptolide (TPL) is a natural product isolated from the traditional Chinese herb which possesses potent anti-tumor activity in human cancers. However, its role in pyroptosis remains to be elucidated.

**Methods:**

Cell survival was measured by colony formation assay. Cell apoptosis was determined by Annexin V assay. Pyroptosis was evaluated by morphological features and release of interleukin 1β and lactate dehydrogenase A (LDHA). Immunofluorescence staining was employed to measure subcellular localization of proteins. Tumorigenicity was assessed by a xenograft tumor model. Expression levels of mRNAs or proteins were determined by qPCR or western blot assay, respectively.

**Results:**

Triptolide eliminates head and neck cancer cells through inducing gasdermin E (GSDME) mediated pyroptosis. Silencing GSDME attenuates the cytotoxicity of TPL against cancer cells. TPL treatment suppresses expression of c-myc and mitochondrial hexokinase II (HK-II) in cancer cells, leading to activation of the BAD/BAX-caspase 3 cascade and cleavage of GSDME by active caspase 3. Silencing HK-II sensitizes cancer cells to TPL induced pyroptosis, whereas enforced expression of HK-II prevents TPL induced pyroptosis. Mechanistically, HK-II prevents mitochondrial translocation of BAD, BAX proteins and activation of caspase 3, thus attenuating cleavage of GSDME and pyroptosis upon TPL treatment. Furthermore, TPL treatment suppresses NRF2/SLC7A11 (also known as xCT) axis and induces reactive oxygen species (ROS) accumulation, regardless of the status of GSDME. Combination of TPL with erastin, an inhibitor of SLC7A11, exerts robust synergistic effect in suppression of tumor survival in vitro and in a nude mice model.

**Conclusions:**

This study not only provides a new paradigm of TPL in cancer therapy, but also highlights a crucial role of mitochondrial HK-II in linking glucose metabolism with pyroptosis.

**Supplementary Information:**

The online version contains supplementary material available at 10.1186/s13046-021-01995-7.

## Background

Pyroptosis is a pro-inflammatory form of regulated cell death. It is characterized by cell swelling and rapid cell rupture, followed by release of pro-inflammatory interleukin (IL)-1β, IL-18 and other cellular contents to extracellular space [1, 2]. Pyroptosis was originally described to occur in Salmonella infected macrophages and is accompanied by caspase-1 activation [3–5]. In addition to bacterial infection, non-bacterial pyroptosis also occurs in epithelial cells upon various death stimuli. For example, chemotherapy drugs induce pyroptosis in intestinal epithelial cells [6]. Recent studies unveiled that targeted therapy and chemotherapy eliminate cancer cells through induction of pyroptosis in cancer cells [7–9]. Pyroptosis has attracted growing attention because of its potential to increase the efficacy of cancer immune therapy [10]. Pyroptosis is executed by gasdermins, a pore-formation proteins family [11]. The gasdermins family proteins contain a pore-forming N-terminal domain and a self-inhibitory C-terminal domain connected by a flexible linker. The caspases cleave gasdermins in its linker and liberate the cytotoxic N-terminal domain, which initiates pyroptosis by forming pores in cell membrane [12–14]. Gasdermin E (GSDME) is a potent tumor suppressor and has the potential to evoke anti-tumor immunity through mediating pyroptosis in cancer cells [15].

Triptolide (TPL, C20H24O6, molecular weight: 360.4) is a natural diterpenoid epoxide within potent anti-cancer activity [16–18]. TPL is one of the major bioactive ingredients of the Chinese traditional Herb *Tripterygium wilfordii Hook f* (TWHf), which has been used to treat inflammatory, autoimmune and malignant diseases more than hundreds years [19]. TPL or its derivatives possess potent anti-tumor activity in a variety of human cancers [18]. To date, several TPL derivatives are currently in phase I/II clinical trial for cancer treatment [18]. The anti-cancer activity of TPL has been linked to activation of apoptosis signaling [20]. However, the mechanisms underlying tumor suppressive activity of TPL remain to be fully explored.

In this study, we demonstrated that TPL treatment robustly eliminated head and neck cancer cells through GSDME-dependent pyroptosis. TPL suppressed mitochondrial HK-II and aerobic glycolysis, consequently leading to mitochondrial translocation of BAX/BAD complex and cleavage of GSDME by active caspase 3. Furthermore, TPL suppressed NRF2/SLC7A11 axis and synergized with erastin to kill both GSDME-expressing and GSDME-deficient cancer cells.

## Methods

### Cell lines, cell culture, and transfection

HK1 is a differentiated squamous cell carcinoma cell line from nasopharynx carcinoma maintained in our lab [21]. C666–1 is an undifferentiated nasopharyngeal carcinoma cell line maintained in our lab [22]. FaDu is a hypopharyngeal carcinoma cell line purchased from the National Infrastructure of Cell Line Resource (Shanghai, China) [23]. All these cells were cultured in RPMI 1640 medium (Gibco, Grand Island, NY, USA) containing 10% fetal bovine serum (FBS, Gibco) and 1% penicillin-streptomycin. All cell lines were maintained in a humidified atmosphere consisting of 5% CO_2_ and 95% air at 37 °C.

TPL (Cat.#T3652, Sigma-Aldrich) and erastin (Cat.#S7242, Selleck, China) powders were dissolved in dimethyl sulfoxide (DMSO, Sigma-Aldrich) to achieve a 1 mM or 10 mM stock solution, respectively. The final DMSO concentration in the medium did not exceed 0.1% (volume per volume). MG132 and cycloheximide (CHX) were purchased from Sigma. MG132 (10 mM), CHX (50 mg/mL) were dissolved in DMSO as stock solutions, respectively. The stock solutions were kept in aliquot at − 20 °C and thawed immediately before each experiment.

### MTT (3- (4,5-dimethylthiazol-2-yl)-2,5-diphenyltetrazolium bromide) assays

MTT assays were performed according to previously described [24, 25]. Briefly, cells (5 × 10^4^ cells/mL) were plated in 96-well plates and treated with different dose of TPL for 24 h or 48 h. Cells were incubated with 10 μl of MTT (5 mg/ml) for 4 h to form purple formazan crystals. Then purple formazan crystals were dissolved by 100 μl of DMSO. The absorbance of sample was measured at 490 nm using a microplate reader. Data were presented as means ± SD from five wells per experiment.

### Colony formation assay

HK1, FaDu and C666–1 cells were plated in 6-well plates at a density of 2 × 10^3^ cells/well. After 24 h, the medium was changed to fresh complete medium including the indicated dosage of TPL. Cells were treated with different dose of TPL or erastin for 24 h and then cultured with fresh medium for two weeks. Colonies were fixed with 4% paraformaldehyde and visualized by 0.1% crystal violet according to previous demonstration [26, 27].

### Cell apoptosis assay

Apoptotic cells were determined by using FITC Annexin V Apoptosis Detection Kit (Cat.#BA11100, Enogene, China) according to the manufacturer’s protocol. Briefly, single cell suspension was incubated with 3 μl annexin V-FITC and 3 μl propidium iodide (PI) for 10 min incubation at room temperature. Cells were analyzed by flow cytometry (FACS Calibur, BD, USA). A total of 10,000 events were recorded for each analysis using FlowJo software.

### RNA extraction and real-time reverse-transcription PCR (qPCR)

Total cellular RNAs were extracted by using the Trizol reagent (Life Technologies, Grand Island, NY). After digested with RNase-free DNase I (Takara, Beijing, China), complementary DNA was prepared by using a RevertAid First Strand cDNA Synthesis Kits (Thermo Fisher Scientific, Beijing, China). SYBR Green (Bimake, Shanghai, China)-based quantitative PCR was performed by using the CFX96 Touch™ Real-Time PCR Detection System (Bio-Rad, Richmond, CA, USA). The relative expression levels of genes were counted according to 2^-ΔΔCT^ methods. Primers used in the qPCR assays were shown in Supplementary Table S1.

### RNA-Seq

RNA samples were prepared as described above. Sequencing was performed by using an Illumina HiSeq platform (San Diego, CA, USA). Differentially expressed genes with fold change ≥2 were determined by NOISeq method [28]. Gene set enrichment analysis (GSEA) was performed as described previously [29].

### Western blot analysis

Cells were lysed with RIPA buffer and the supernatants were collected by centrifuge as described previously [30]. Cellular proteins (50 μg/sample) were separated by 10% SDS-PAGE and transferred to PVDF membranes (Millipore, Billerica, MA, USA). PVDF membranes were blocked with 5% non-fat milk for 1 h and then incubated with primary antibodies at 4 °C overnight. The membranes were washed three times (10 min per wash) with washing buffer and then incubated with a secondary antibody at 37 °C for 1 h. Immunostaining was developed by using the ECL detection system (Thermo Fisher Scientific) and recorded by Bio-Rad ChemiDoc XRS. All primary antibodies used in this study were listed in the Supplementary Table S2.

### Immunofluorescence assay

Cells were fixed with 4% paraformaldehyde at 37 °C for 60 min and then permeabilized by 0.1% Triton X-100 for 10 min. Cells were blocked with 5% bovine calf serum for 30 min at room temperature and then incubated with indicated primary antibodies at 4 °C overnight. Fluorescence was developed by incubating with Cy3 or FITC labeled goat anti-rabbit IgG for 1 h at 37 °C. The nucleus was counterstained with DAPI. Fluorescence image were captured under a laser confocal fluorescence microscope (Olympus).

### siRNAs transfection and shRNA

GSDME, BAX, BAD, caspase 3, c-myc and HK- II in cancer cells were transiently silenced by transfection of targeted siRNAs using Lipofectamin™ RNAiMAX (Invitrogen, Carlsbad, CA) according to the manufacturer’s instructions. For stable silencing, cells were infected with shRNA expressing lentivirus and selected by puromycin. For stable overexpression, cells were infected with GSDME, HK- II and c-myc expressing lentivirus and selected by puromycin. All siRNAs used in this study were purchased from GenePharma Co., Ltd. (Shanghai, China). Sequences of siRNAs or shRNAs were listed in Supplementary Table S3.

### Measurements of glucose consumption and lactate production

Cells were cultured for indicated times and the culture mediums were collected and centrifuged to remove any suspended cells. Glucose concentration in culture medium was detected by using a Glucose Assay Kit (Cat.#361,510, rsbio, China) according to the manufacturer’s instructions. Glucose consumption was assessed by decrease of glucose concentration as compared to fresh culture medium. Lactate in culture mediums was measured by using a lactic acid assay kit (Cat.#A019–2, Nanjing Jiancheng Bioengineering Institute, China) according to the manufacturer’s instructions.

### Cellular ATP assay

Cells were pre-incubated with 50 nM TPL for 24 h. Adenosine triphosphate (ATP) contents in cells were measured with an ATP Colorimetric/Fluorometric Assay Kit (Cat. #K354, BioVision, USA) in accordance with the manufacturer’s instructions.

### ELISA (enzyme-linked immunosorbent assay) and LDH1 activity assays

Cells were treated with TPL for indicated times. Culture mediums were collected and centrifuged to remove any cell debris or suspended cells. IL-1β released into culture medium was measured by using a Human IL-1β ELISA Set II (Cat. RUO – 557,953, BD Biosciences,US). LDH1 activity in culture medium was measured by using a LDH1 activity assay kit (Cat. #88954, Thermo scientific, Rockford, IL, USA). The protein levels of LDHA released into culture medium were determined by western blot assays.

### ROS detection

Intracellular ROS was labeled by the fluorescent probe 2′,7′-dichlorofluorescin diacetate (DCFH-DA) (Cat. #C400, Invitrogen, Carlsbad, CA) and then was analyzed by flow cytometry.

### Xenograft tumor formation assays and in vivo imaging

Single cell suspension (1 × 10^6^ cells/0.2 mL) was subcutaneously injected into the 5-week-old male BALB/c nude mice (Shanghai SLAC Laboratory Animal Co. Ltd., Shanghai, China). After 2 weeks of transplantation, 20 mice were randomly assigned to the following 4 groups, (1) control group: mice were given intraperitoneal injection i.p.of 0.1% DMSO in saline. (2) TPL group: mice were treated with 0.1 mg/kg TPL i.p. for one injection, (3) combination of TPL and erastin group: mice were treated with 10 mg/kg erastin i.p. followed by 0.1 mg/kg TPL, (4) erastin group: mice were treated with 10 mg/kg erastin i.p.injection. The tumor volumes were monitored using a digital caliper. Xenograft tumor volume (mm^3^) was calculated as the following formula: 0.5 × (the shortest diameter)^2^ × (the longest diameter). After treatment with TPL for 10 days, the mice were intraperitoneally injected with a luciferin solution (Sigma-Aldrich, Beijing, China) at a dose of 150 mg/kg (15 mg/ml in PBS). Tumors were evaluated once by using the IVIS® Lumina System (Xenogen Corporation, California, USA). The mice were sacrificed 10 days after drugs administration. The dissected xenografts were weighed and fixed in 4% saline-buffered formalin, embedded in paraffin, sectioned at 4 μm. Tissue sections were stained with hematoxylin and eosin (H&E) or analyzed by immunohistochemistry staining. All of the animal experimental procedures in this study were performed in accordance with the protocol approved by Animal Welfare Committee of Central South University.

### Statistical analysis

Student’s t-test was employed to analyze the quantitative variables differences between groups. All results were expressed as mean ± SD. Xenograft tumor growth between different groups was analyzed by a two-way ANOVA. Statistical analysis was performed by using the SPSS v17.0 software package (SPSS, Chicago, IL, USA). A value of *P* < 0.05 was considered as statistically significant. Statistical histograms were performed using Prism 5.0 (Graphpad Software, CA, USA) and SPSS v17.0 software (SPSS, Chicago, IL, USA).

## Results

### TPL induced pyroptotic cell death in head and neck cancer cells

**Fig. 1:**
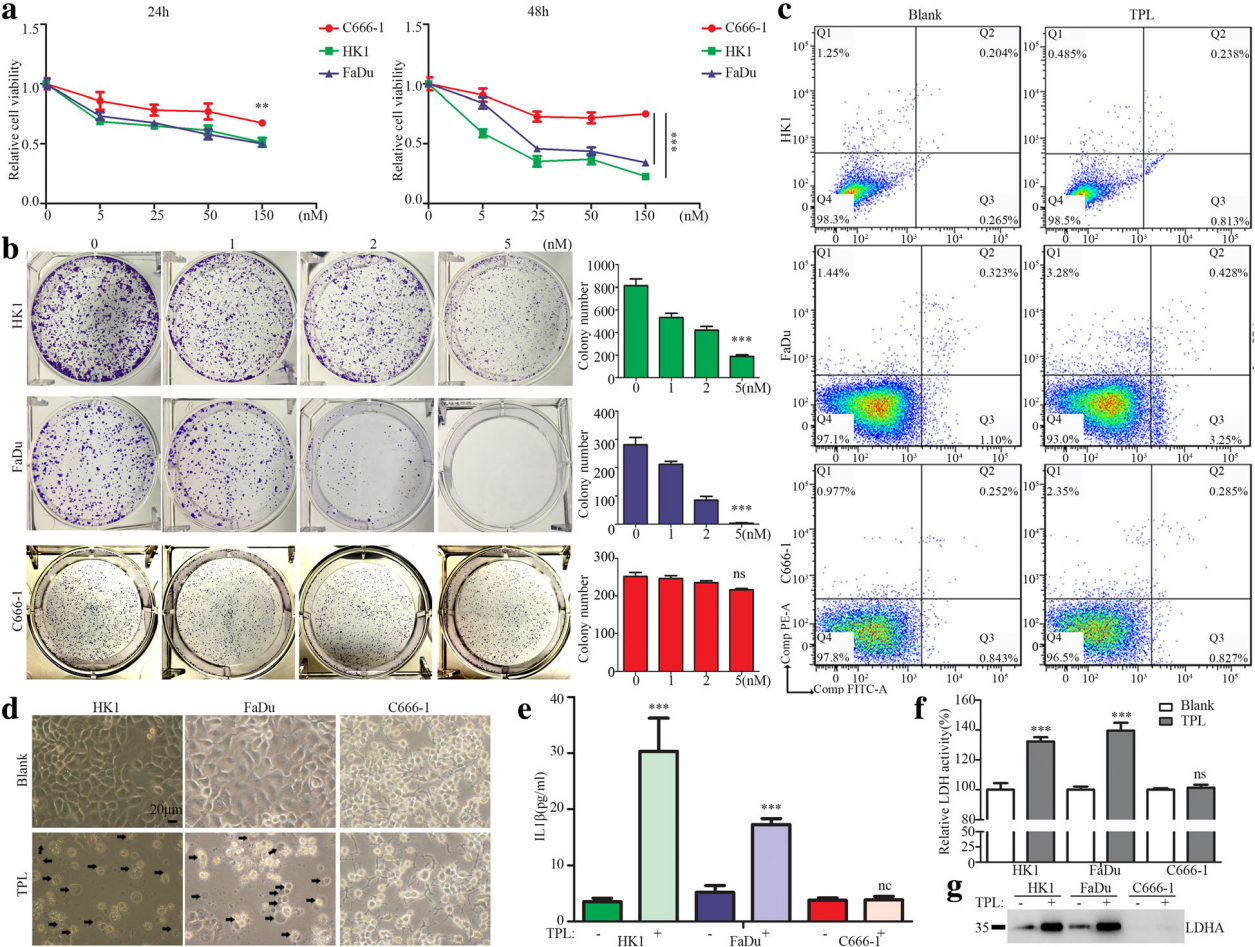
TPL induces pyroptosis in head and neck cancer cells. **a** Cell viability assays. HK1, FaDu and C666–1 cells were exposed to various concentrations of TPL (0, 5, 25, 50 and 150 nM) for 24 h or 48 h. Cell viability were determined by MTT assays. **b** Colony formation assays. Cells were treated with various doses of TPL (0, 1, 2, and 5 nM) for 48 h and then allowed to grow for 2 weeks in fresh culture medium. Colonies were visualized by crystal purple staining. Values are mean ± SD from triplicate experiments. **c** Apoptotic cell frequencies treated without or with TPL (50 nM) for 24 h were determined by annexin V/PI assays. **d** Morphological alterations induced by TPL (50 nM) treatment. Arrow showed cell swelling and rupture. **e** IL-1β released into culture medium was detected by ELISA assays. **f** LDH1 activity assays. LDH1 activity in culture mediums of cells treated without or with TPL (50 nM) for 24 h were measured by LDH1 kit. **g** Release of LDHA proteins to culture mediums were detected by western blot assays. *P < 0.05, **P < 0.01, ***P < 0.001

To assess the effect of TPL on head and neck cancer cell lines, we initially treated diverse cancer cell lines with 0–150 nM TPL. MTT assay showed TPL remarkably suppressed cell viability of HK1 and FaDu cells in a dose- and time-dependent manner (Fig. 1a). TPL also exerted suppressive effect on the C666–1 cell, which is an EBV-positive nasopharyngeal carcinoma cell line. However, as compared to HK1 and FaDu cells, C666–1 cell is more refractory to TPL (Fig. 1a). Colony formation assay revealed that TPL dramatically reduced colony numbers of HK1 and FaDu cells in a dose-dependent manner. However, it is not the case in C666–1 cell (Fig. 1b). Annexin V/PI staining assays revealed that TPL treatment did not cause apoptosis in HK1, FaDu and C666–1 cells (Fig. 1c). However, we observed robust cell death in HK1, FaDu cells but not in C666–1 cell upon TPL treatment under microscope (Fig. 1d). HK1, FaDu cells treated with TPL displayed cytoplasmic swelling and membrane rupture, whereas C666–1 cells remained viable despite of reduced cell confluency upon TPL treatment (Fig. 1d). Morphological alterations in HK1, FaDu cells suggested pyroptosis occurred after TPL treatment. ELISA assays indicated that dramatic release of IL-1β cytokine into the culture mediums of HK1, FaDu cells treated with TPL, but not in C666–1 cell (Fig. 1e). LDH1 activity assays revealed increased LDH1 activity in culture mediums of HK1, FaDu cells treated with TPL, but not in C666–1 cell (Fig. 1f). Western blot assays also demonstrated that abundant LDHA proteins presented in culture mediums of HK1, FaDu cells but not in that of C666–1 cell treated with TPL (Fig. 1g). Thus, morphological and biochemical features clearly indicated TPL selectively induced pyroptosis in head and neck cancer cells HK1 and FaDu.

### TPL induced cancer cells pyroptosis is mediated by GSDME

**Fig. 2:**
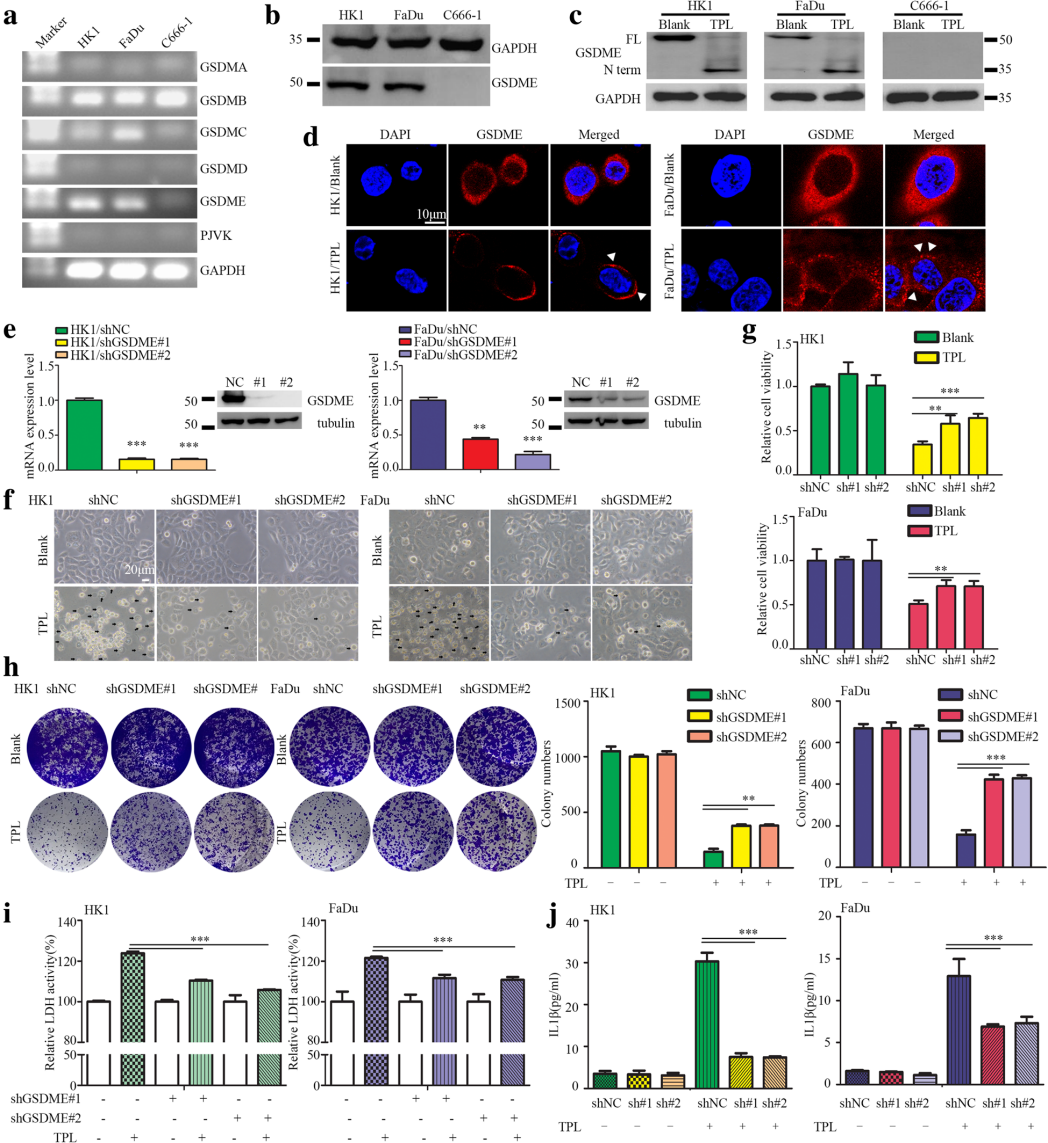
TPL induced pyroptosis in head and neck cancer cells is executed by GSDME. **a** The mRNA levels of gasdermin members were determined by RT-PCR assays. **b** The protein levels of GSDME were measured by western blot assays. **c** The Protein levels of full-length GSDME and GSDME-N terminus in cells treated without or with TPL (50 nM) for 24 h were measured by western blot assays. **d** GSDME proteins localization in cells treated without or with TPL (50 nM) for 24 h were visualized by immunofluorescence assays. **e** The mRNA or protein levels of GSDME in shRNAs expressing lentivirus infected cells were measured by qPCR or western blot. **f** Morphological features of cells treated with TPL showed reduced pyroptotic cell death in GSDME-silenced cells. Cells were treated with TPL (50 nM) for 24 h. **g** Cell viability was measured by MTT assay. **h** Cell survival was measured by colony formation assay. Cells were treated with TPL (10 nM) for 24 h. **i** LDH1 activity assays. **j** IL-1β released into culture medium was detected by ELISA assays. Values are shown as mean ± SD from triplicate experiments. *P < 0.05, **P < 0.01, ***P < 0.001

We measured the mRNA levels of gasdermins genes in head and neck cancer cells used in this study. RT-PCR assays revealed that the mRNA levels of GSDMA, GSDMD and PJVK were undetectable in HK1, FaDu and C666–1 cells, whereas GSDMB was consistently expressed. GSDMC was selectively expressed in FaDu cell but weak in HK1 and C666–1 cells. The mRNA levels of GSDME were highly expressed in HK1 and FaDu cells but not in C666–1 cell (Fig. 2a). Western blot assay showed GSDME protein levels were high in HK1 and FaDu but were undetectable in C666–1 cell (Fig. 2b). TPL treatment resulted in cleavage of GSDME in HK1, FaDu cells (Fig. 2c). Immunofluorescence assays showed that GSDME proteins were diffusely distributed in cytoplasm of HK1 and FaDu cells prior TPL treatment, whereas appeared membrane localized puncta after TPL treatment (Fig. 2d), suggesting pores formation of GSDME-N terminal in membrane after TPL treatment. We then exploited shRNAs expressing lentivirus to silencing GSDME expression. RT-PCR and western blot assays showed that two shRNAs targeted to GSDME effectively reduced its mRNA and protein levels in HK1, FaDu cells (Fig. 2e). Inhibition of GSDME prevented TPL induced pyroptotic cell death in HK1, FaDu cells, as evidenced that less of cytoplasmic swelling and membrane rupture in GSDME-silenced cells when treated with TPL (Fig. 2f). MTT assays also showed silencing GSDME preserved cell viability in HK1 and FaDu cells upon TPL treatment (Fig. 2g). Colony formation assays showed that loss of GSDME promoted cancer cells survival upon TPL treatment (Fig. 2h). We also found that there were less TPL-induced release of IL-1β and LDH1 into culture mediums when GSDME were silenced in HK1, FaDu cells (Fig. 2i&j). Thus, our data suggested that TPL induced pyroptosis in HK1 and FaDu cells are mainly mediated by GSDME.

### Activation of BAD/BAX-caspase 3 cascade is required for GSDME-mediated pyroptosis induced by TPL

**Fig. 3:**
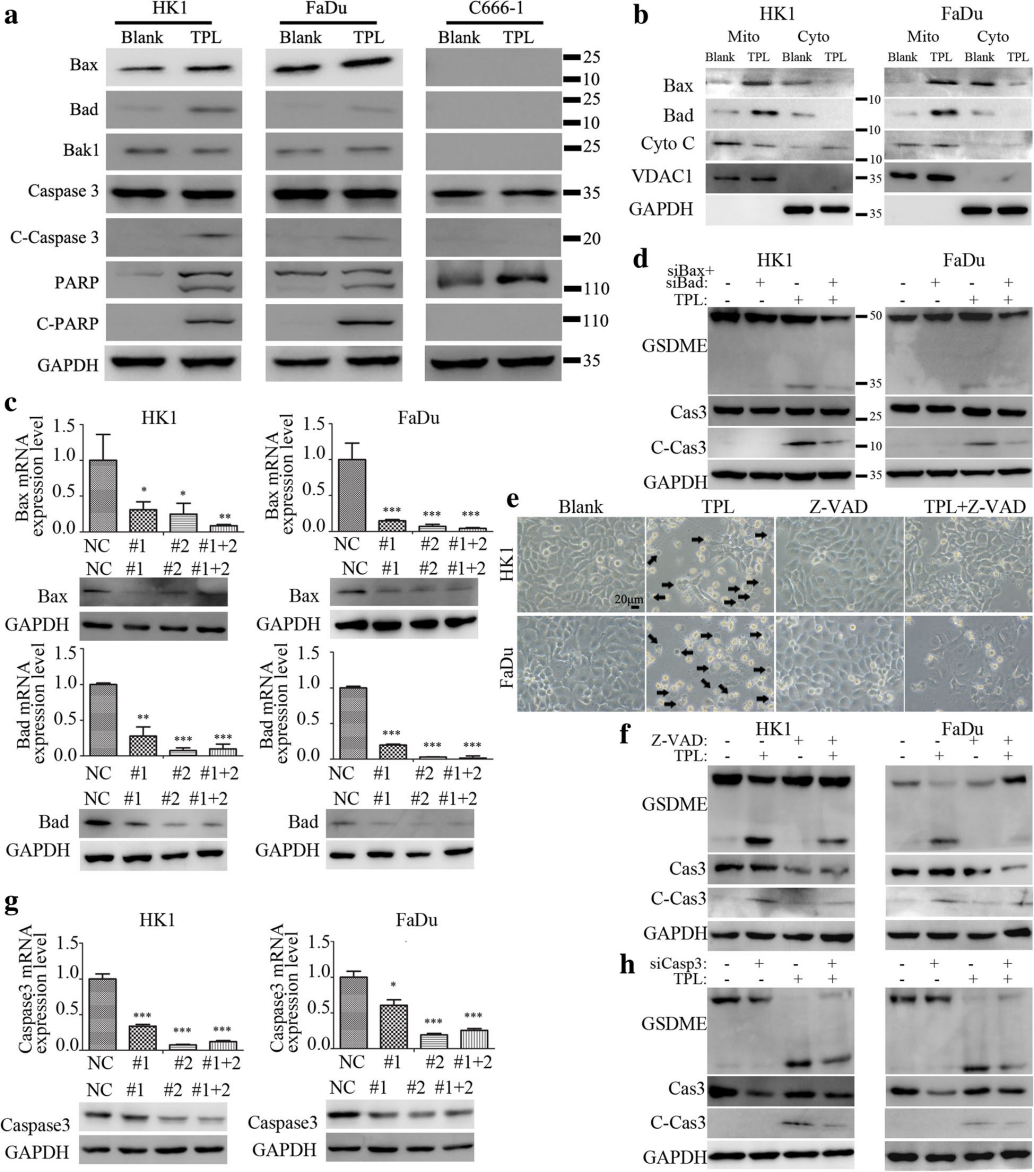
Activation of mitochondrial pathway and caspase 3 are required for cleavage of GSDME and pyroptosis induced by TPL. **a** The protein levels of mitochondrial death pathway were determined by western blot. **b** sub-cellular distributions of BAX, BAD and cytochrome c proteins were determined by subcellular fraction and western blot assays. **c** The mRNA and protein levels of BAX, BAD in siRNAs transfected cells were determined by qPCR or western blot. **d** Cleavage of caspase 3 and GSDME in BAX/BAD-depleted cells were measured by western blot. **e** Morphological features showed less cell swelling and rupture induced by TPL when caspase activity was blocked by z-VAD. **f** Effects of z-VAD on cleavage of GSDME was measured by western blot. **g** The mRNA and protein levels of caspase 3 in siRNAs transfected cells were determined by qPCR or western blot. **h** Cleavage of GSDME in caspase 3 silenced cells were determined by western blot

We then asked the upstream modulators of GSDME in TPL induced pyroptosis. Western blot assays showed that TPL treatment upregulated the protein levels of BAX, BAD in HK1 and FaDu cells, without affecting the protein level of BAK1. TPL treatment also resulted in activation of caspase 3 in HK1 and FaDu cells, as evidenced by cleavage of caspase 3 and PARP (Fig. 3a). However, there were no BAX, BAD and BAK1 proteins detected in C666–1 cell, regardless of TPL treatment. No cleaved caspase 3 and cleaved PARP were detected in C666–1 cell treated with TPL (Fig. 3a). Subcellular fraction assays revealed that TPL treatment promoted mitochondrial translocation of BAD and BAX proteins, consequently led to release of cytochrome c into cytoplasm (Fig. 3b). We used specific siRNAs to silencing BAD or BAX in HK1, FaDu cells. RT-PCR and western blot assays demonstrated that transient transfection with siRNAs efficiently suppressed the mRNA and protein levels of BAD, BAX in HK1, FaDu cells (Fig. 3c). Simultaneously inhibition of BAD and BAX attenuated activation of caspase 3 and cleavage of GSDME in HK1, FaDu cells upon TPL treatment (Fig. 3d). Furthermore, z-VAD, a pan-caspase inhibitor of caspases, blocked TPL induced pyroptotic cell death in HK1, FaDu cells (Fig. 3e). Western blot assay suggested that z-VAD prevented activation of caspase 3 and inhibited cleavage of GSDEM in TPL treated cells (Fig. 3f). More specifically, we depleted caspase 3 expressions in HK1, FaDu cells by transfection of siRNAs. RT-PCR and western blot assays showed specific siRNAs successfully reduced the mRNA and protein levels of caspase 3 (Fig. 3g). Consequently, loss of caspase 3 impeded cleavage of GSDME in HK1, FaDu cells induced by TPL (Fig. 3h). Thus, these data clearly suggested that activation of BAD/BAX-caspase 3 cascade is required for cleavage of GSDME and induction of pyroptosis upon TPL treatment.

### TPL treatment repressed c-Myc/HK-II axis and aerobic glycolysis in head and neck cancer cells

**Fig. 4:**
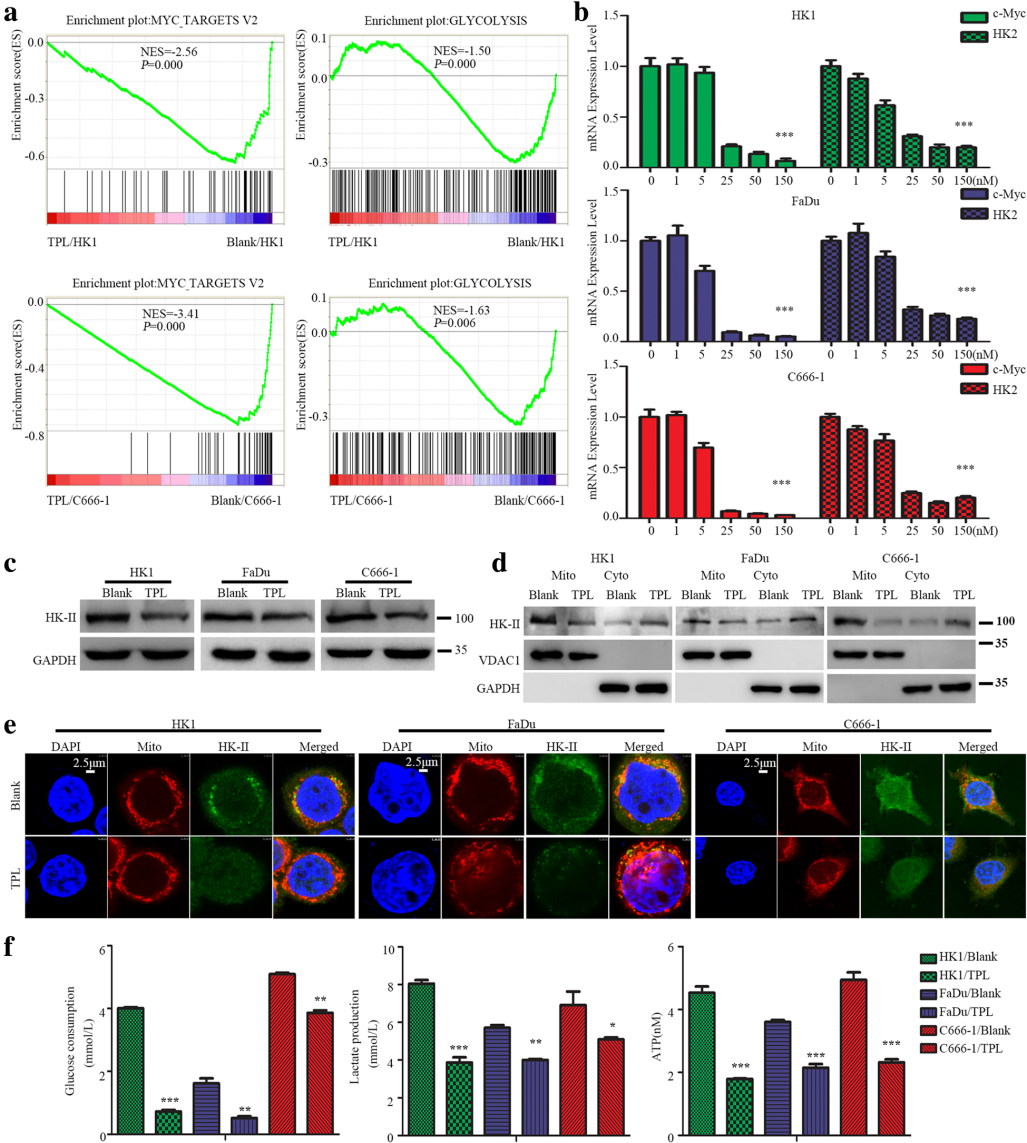
TPL suppresses mitochondrial HK-II and glycolysis in head and neck cancer cells. **a** GSEA analysis indicated TPL induced transcriptomic alterations were associated with c-myc targets and glycolysis. **b** qPCR assay showed that TPL suppressed c-myc, HK-II mRNA levels in head and neck cancer cells in a dose-dependent manner. **c** The protein levels of HK-II were measured by western blot. **d** distribution of HK-II upon TPL treatment was determined by subcellular fraction and western blot. **e** co-localization of HK-II with mitochondria was assessed by immunofluorescence assay. **f** glycolytic rates were assessed by glucose consumptions, lactate productions and cellular ATP contents assays. *P < 0.05, **P < 0.01, ***P < 0.001

To explore possible upstream mechanisms underlying TPL induced pyroptotic cell death in cancer cells, we employed RNA-seq to study TPL induced transcriptomic alterations in HK1 and C666–1 cells. Totally, there were 2361 genes consistently up-regulated, whereas 2236 genes consistently down-regulated by TPL in both HK1 and C666–1 cells (Supplementary materials S1). GSEA analysis indicated that genes downregulated by TPL were functionally enriched in processes related to c-myc targets and glycolysis (Fig. 4a). RT-PCR and western blot assays demonstrated that TPL (50 nM) treatment for 24 h repressed more than 90% of c-myc mRNA and protein in HK1, FaDu and C666–1 cells (Fig. S1A&B). Immunofluorescence assay also revealed TPL treatment totally abolished nuclear staining of c-myc protein in HK1 cell (Fig. S1C). Pulse-chase assay suggested that the half-life of c-myc protein in TPL treated HK1 cell was shortened to less than 1 h (Fig. S1D). Degradation of c-myc protein is prior to inhibition of c-myc transcription by TPL treatment, because the mRNA levels of c-myc in HK1 and FaDu cells remained unchanged in the first 2 h within TPL treatment. Since 4 h later after TPL treatment, c-myc mRNA started to decrease rapidly (Fig. S1E). These results suggested that TPL transcriptionally and post-translationally repressed c-myc expression in head and neck cancer cells.

RNA-seq indicated that hexokinase II (HK-II), which is a known target gene of c-myc that catalyzes the first rate-limiting step of glycolysis, was down-regulated by TPL. Silencing c-myc reduced the mRNA level of HK-II in HK1 and FaDu cells, whereas overexpression of c-myc increased the mRNA level of HK-II, indicating that c-myc transcriptionally regulates HK-II in head and neck cancer cell (Fig. S2). RT-PCR assays showed TPL reduced mRNA levels of c-myc and HK-II in a dose-dependent manner (Fig. 4b). Western blot assays showed that TPL (50 nM) treatment suppressed HK-II protein levels in head and neck cancer cells (Fig. 4c). Sub-cellular fraction assays revealed that TPL (50 nM) reduced the levels of mitochondrial bound HK-II protein in HK1, FaDu and C666–1 cells (Fig. 4d). Immunofluorescence assays showed that TPL (50 nM) treatment reduced co-localization of HK-II protein with mitochondria in HK1, FaDu and C666–1 cells (Fig. 4e). Consequently, TPL inhibited aerobic glycolysis of cancer cells, as evidenced by reduced glucose consumption, lactate production and cellular ATP content following TPL treatment (Fig. 4f).

### TPL activated BAD/BAX-caspase 3-GSDME cascade through repressing mitochondrial associated HK-II

**Fig. 5:**
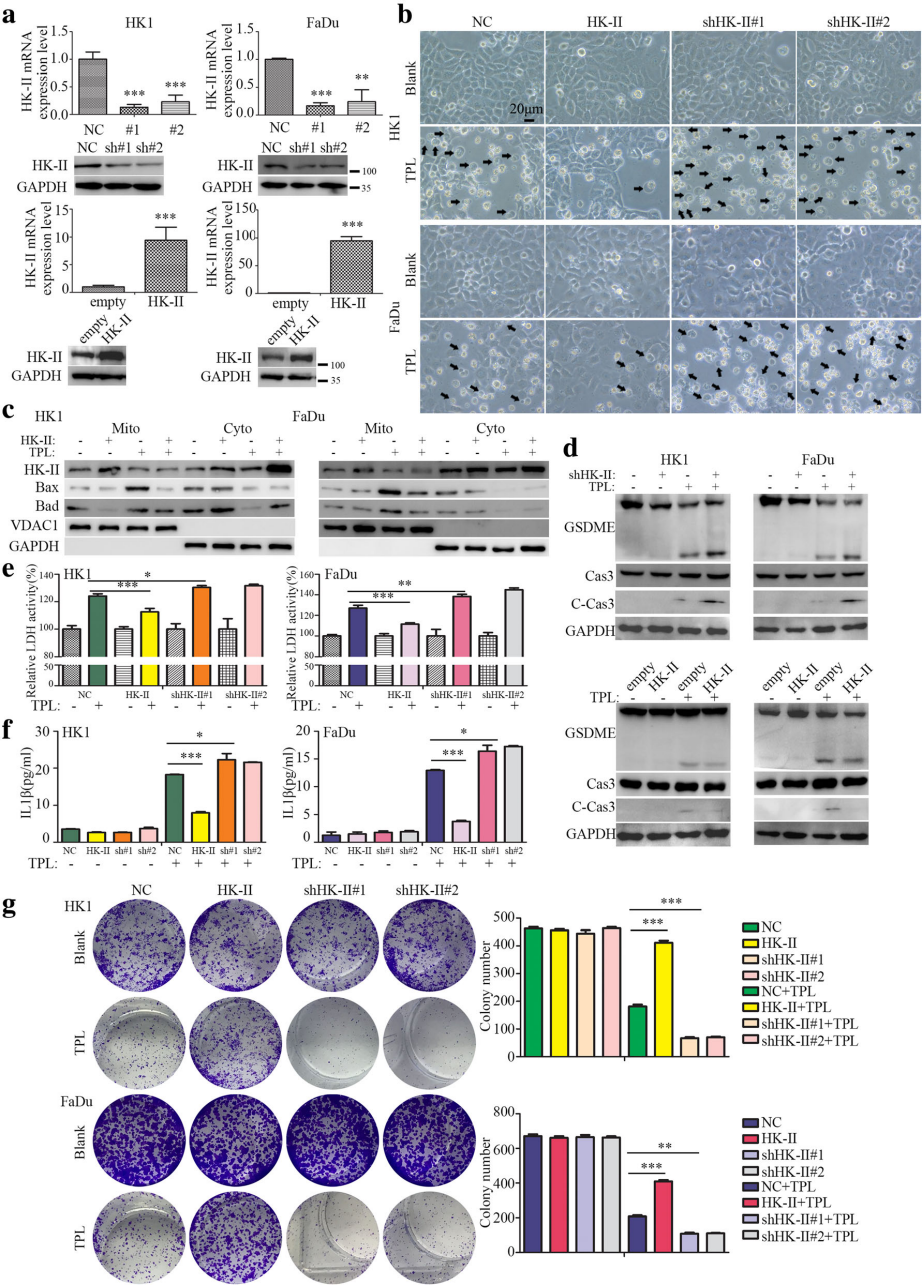
Mitochondrial HK-II prevents TPL induced pyroptosis. **a** The mRNA and protein levels of HK-II in shRNAs or cDNA expressing lentivirus infected cells were assessed by qPCR or western blot. **b** Phase contrast images showed morphological features HK-II silenced or HK-II overexpressed cells upon TPL treatment. **c** Subcellular fraction assays suggested that silencing HK-II promoted mitochondrial translocation of BAX, BAD. **d** Silencing HK-II facilitated activation of caspase 3 and cleavage of GSDME upon TPL treatment, whereas overexpression of exogenous HK-II exerted opposite effects. **e** ELISA assays showed silencing HK-II promoted release of IL-1β into extracellular space, whereas overexpression of exogenous HK-II reduced the release of IL-1β upon TPL treatment. **f** LDH1 activity assays. **g** cell survival of HK-II depleted or HK-II overexpressed cells were assessed by colony formation assays. Values are shown as mean ± SD from triplicate experiments. *P < 0.05, **P < 0.01, ***P < 0.001

Mitochondrial associated HK-II not only plays an essential role in cancer metabolism, but also regulates cell death [31–33]. We asked whether reduction of mitochondrial associated HK-II was involved in TPL induced pyroptosis. Two HK-II targeting shRNAs expressing lentivirus or a HK-II cDNA-encoding lentivirus were introduced into HK1 and FaDu cells. RT-PCR and western blots assays revealed that two HK-II targeting shRNAs successfully reduced HK-II mRNA and protein levels, whereas HK-II cDNA-encoding lentivirus upregulated HK-II mRNA and protein levels in HK1 and FaDu cells (Fig. 5a). Stable depletion of HK-II was not sufficient to trigger pyroptosis of cancer cells (data not shown), but promoted TPL induced pyroptosis. In contrast, forced expression of HK-II prevented pyroptosis induced by TPL in HK1 and FaDu cells (Fig. 5b). Sub-cellular fraction assays demonstrated that TPL treatment promoted mitochondrial translocation of BAD, BAX proteins, but forced expression of HK-II significantly reduced mitochondrial localized BAD and BAX protein levels upon TPL treatment (Fig. 5c). Silencing HK-II facilitated activation of caspase 3 and cleavage of GSDME in TPL treated HK1 and FaDu cells, whereas overexpression of HK-II exerted opposite effects (Fig. 5d). Consequently, stable depletion of HK-II led to more release of IL-1β and LDH1 into culture medium upon TPL treatment, whereas forced expression of HK-II exerted opposite effects (Fig. 5e&f). Colony formation assays also showed silencing HK-II severely impaired cell survival, whereas overexpression of HK-II enhanced cell survival upon TPL treatment (Fig. 5g). Thus, our data suggested that TPL treatment activated BAD/BAX-caspase 3-GSDME cascade through repressing mitochondrial associated HK-II.

### TPL in combination with erastin inhibits SLC7A11 expression, increases sensitivity to ferroptosis

**Fig. 6:**
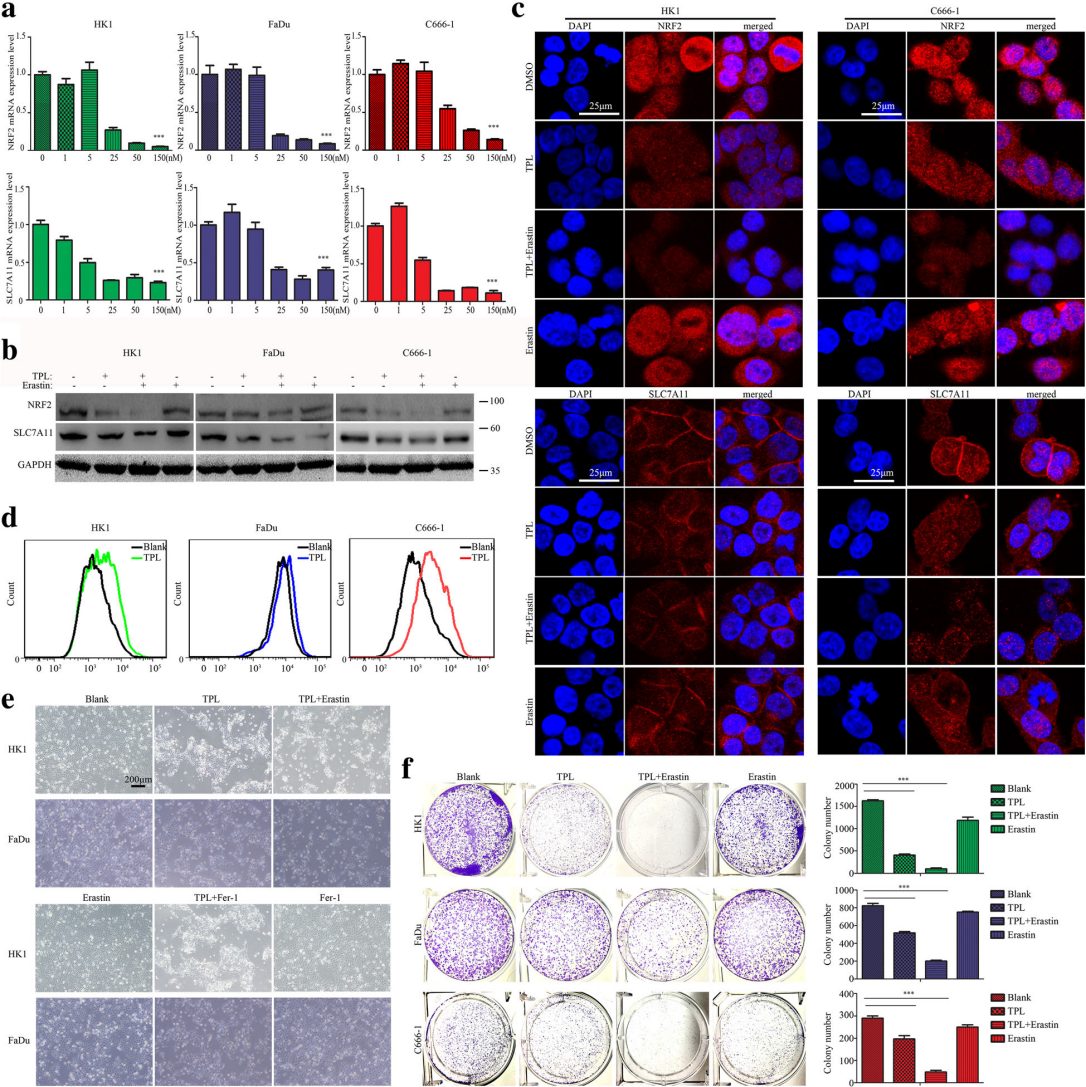
TPL suppresses NRF2/SLC7A11 axis and synergizes with erastin to eliminate both GSDME-expressing and GSDME-deficient cancer cells. **a** qPCR assays showed TPL suppressed the mRNA levels of NRF2 and SLC7A11 in a dose dependent manner. **b** The protein levels of NRF2 and SLC7A11 were measured by western blot. **c** Immunofluorescence assays of NRF2 and SLC7A11 proteins. Cells were treated with or without 50 nM TPL in the absence or presence of erastin (10 μM) for 24 h. **d** Intracellular ROS were probed with DCFH-DA and measured by FACS. **e** Phase contrast images showed morphological features of cells under indicated conditions. **f** cell survival was assessed by colony formation assays. Data are shown as mean ± S.D. from three independent experiments. *P < 0.05, **P < 0.01, ***P < 0.001, compared with control

As we demonstrated above, C666–1 cell was relatively refractory to TPL induced cell death due to lack of endogenous GSDME expression, despite of that TPL suppressed c-myc/HK-II axis in it. According to RNA-seq, we noticed that TPL treatment also resulted in reduction of NRF2 and its target gene SLC7A11/xCT expression in C666–1 cell along with HK1, FaDu cells. NRF2-SLC7A11/xCT axis is known to plays an essential role in anti-oxidative defense system and prevents lipid oxidation induced ferroptosis [34, 35]. RT-PCR assays revealed that TPL suppressed the mRNA levels of NRF2, SLC7A11 in all these three cancer cell lines in a dose-dependent manner (Fig. 6a). Western blot assays showed that TPL (50 nM) treatment decreased the protein levels of NRF2 and SLC7A11 (Fig. 6b). In addition, combination of TPL with erastin, an inhibitor of SLC7A11, was more efficient to repress the protein level of NRF2 and SLC7A11, as evidenced by western blot and immunofluorescence assays (Fig. 6b&c). We also measured the levels of ROS in cancer cells treated with or without TPL. The results showed that TPL treatment led to accumulation of ROS in HK1, FaDu and C666–1 cells (Fig. 6d). Treatment with erastin (10 nM) alone exerted little effect on cell survival of these cancer cells. However, combination of TPL with erastin more potently eliminated GSDME-expressing HK1, FaDu cells (Fig. 6e). Colony formation assays also revealed that erastin synergized with TPL to kill HK1, FaDu cells (Fig. 6f). It worthy to note that either TPL or erastin alone exerted little effects on survival of C666–1 cells, but these two drugs synergistically killed the C666–1 cancer cell (Fig. 6f), implying TPL and erastin confer a synthetic vulnerability of GSDME-negative cancer cell.

### Combination of TPL and erastin decreased tumorigenicity in a human xenograft model of NPC

**Fig. 7:**
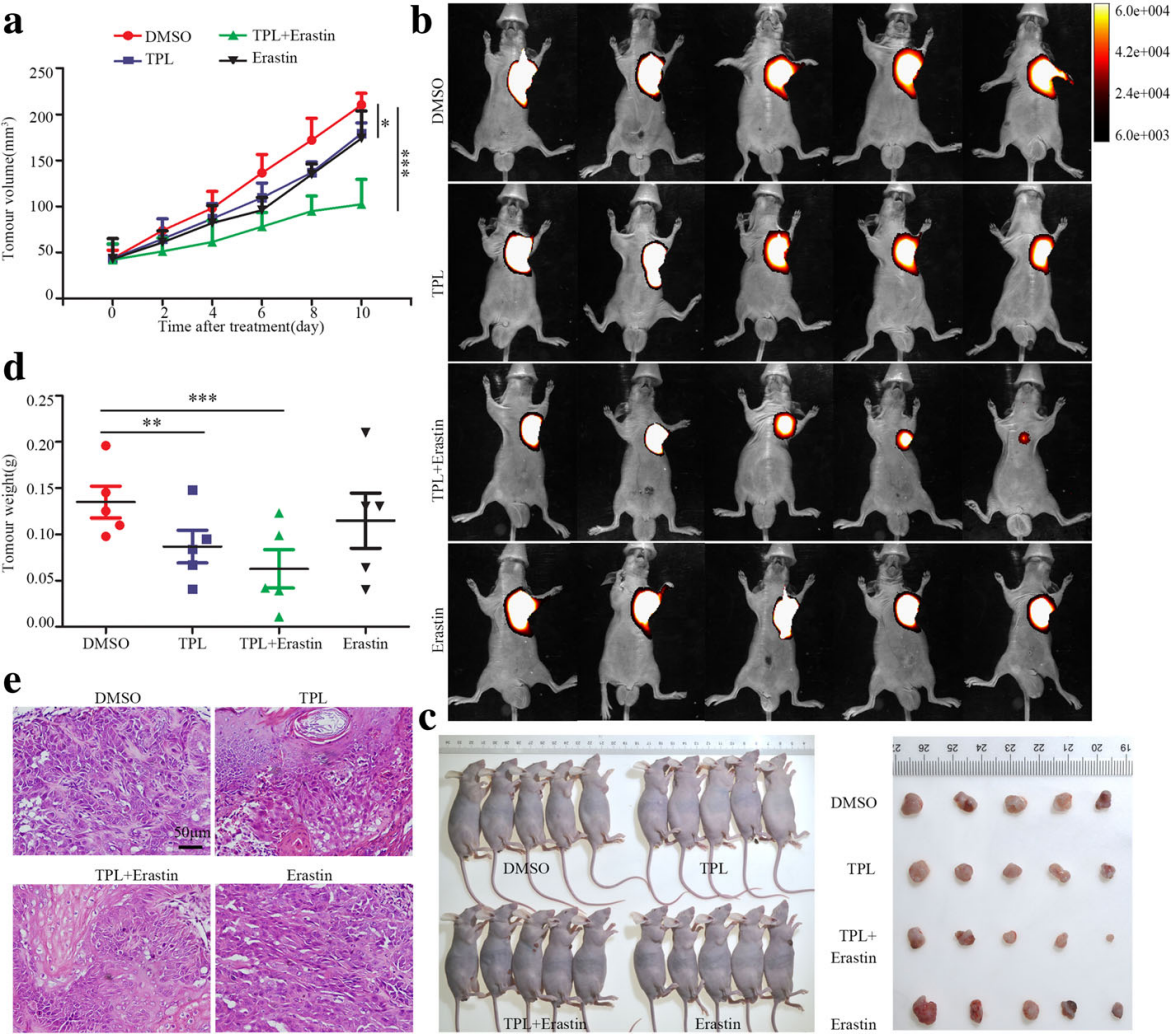
Combination of TPL and erastin potently inhibits tumorigenicity of HK1 cell in vivo. **a** Growth curve of xenograft tumors in mice treated with single reagent or combination of TPL (0.1 mg/kg QD) with erastin (10 mg/kg QD) (*n* = 5/group). **b** Xenograft tumors in live mice were visualized in vivo bioluminescence imaging system. **c** The macroscopic view of mice and xenograft tumors at the endpoint of experiment. **d** The average tumor weight from different groups. Values are shown as mean ± SD (n = 5). **e** H&E staining of xenograft tissue sections. *P < 0.05, **P < 0.01, ***P < 0.001. Scale bar, 50 μm

**Fig. 8:**
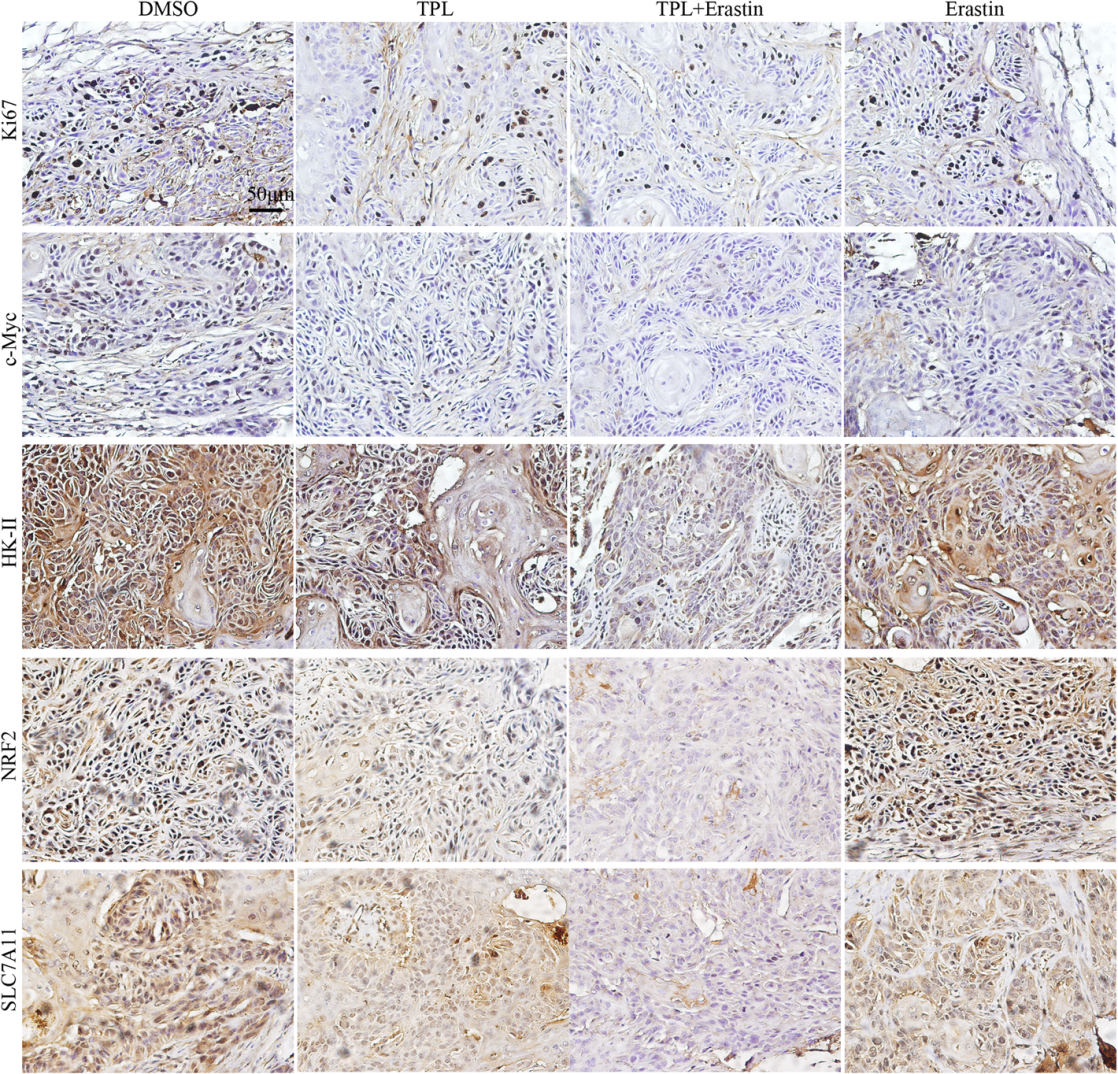
TPL represses the protein levels of Ki67, c-myc, HK-II, NRF2 and SLC7A11 in vivo. The expression levels of Ki67, c-myc, HK-II, NRF2 and SLC7A11 proteins in xenograft tumors were measured by IHC staining. Scale bar, 50 μm

Next, we sought to examine the effect of TPL administration on tumor growth in vivo by using a subcutaneous xenograft tumor model. Our data revealed that TPL or erastin alone moderately decreased tumor growth of HK1 cells, whereas combination of TPL with erastin powerfully inhibited the growth of xenograft tumors in nude mice as compared to single reagents (*P* < 0.01) (Fig. 7a). At the endpoint of the experiment, combination of TPL with erastin significantly reduced the tumor volume and tumor weight as compared to single reagents (Fig. 7b,c&d). H&E staining showed robust necrotic-like death in tumors treated with combination of TPL with erastin (Fig. 7e). Immunohistochemical staining indicated that less Ki-67 positive tumor cells were detected in xenograft tumors from mice treated with combination of TPL and erastin. Also, the protein levels of c-myc, HK-II, NRF2 and SLC7A11 were significantly decreased in tumors from co-treatment group (Fig. 8). These results collectively support the notion that combination of TPL with erastin is a promising strategy for head and neck cancer therapy.

## Discussion

**Fig. 9:**
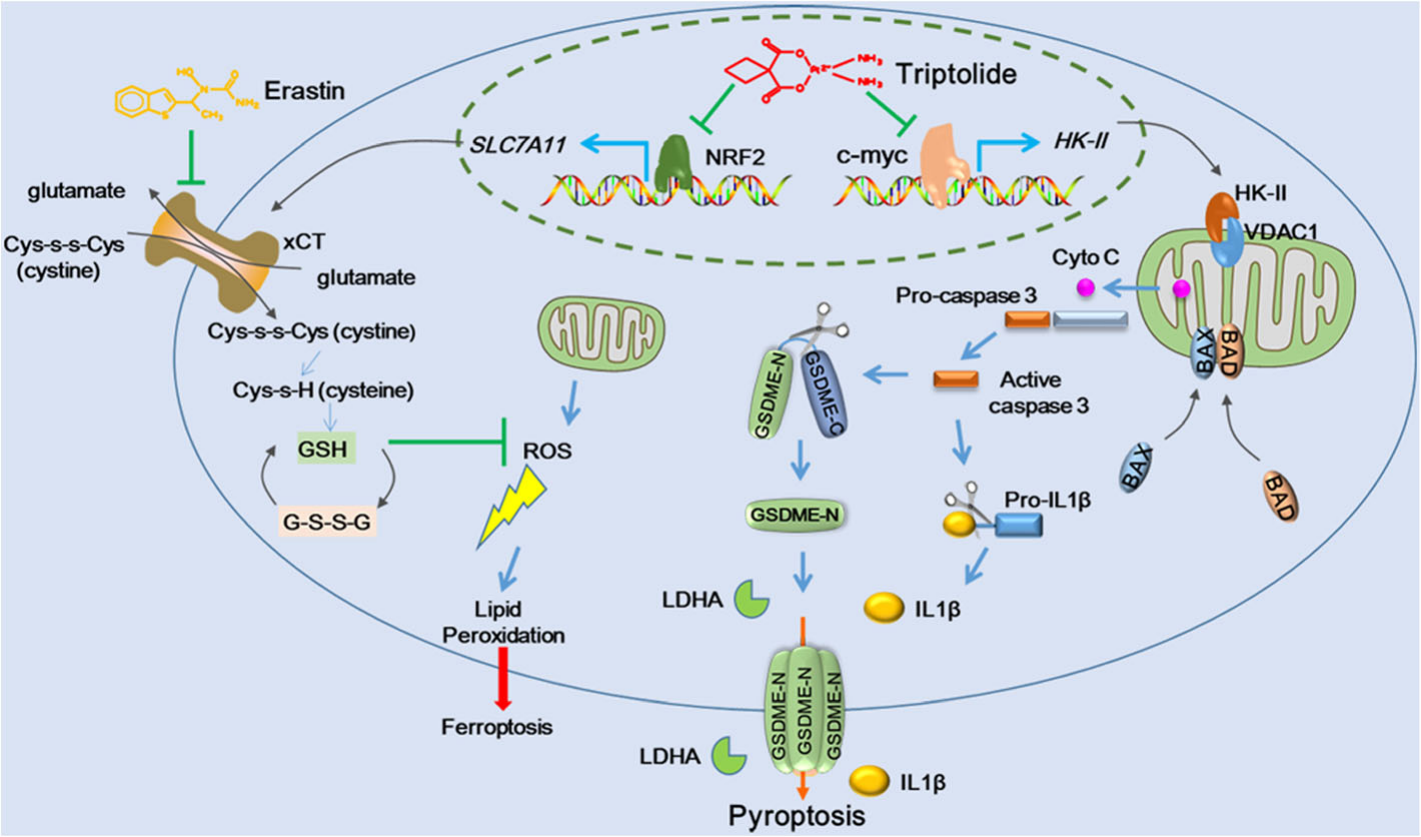
Schematic diagram of mechanisms underlying TPL induced pyroptosis in head and neck cancer cells. TPL simultaneously repressed c-myc/HK-II axis and NRF2/SLC7A11 axis in head and neck cancer cells. Inhibition of mitochondrial HK-II resulted in mitochondrial translocation of BAX and BAD, leading to release of cytochrome c to cytoplasm and subsequent activation of caspase 3. In GSDME ^high^ cancer cells, active caspase 3 cleaved GSDME at internal linker to liberate the pore-forming activity of GSDME-N, resulting in pyroptosis in cancer cells. At the same time, inhibition of NRF2/SLC7A11 by TPL led to accumulation of ROS in cancer cells, thus making cancer cells were sensitive to cytotoxicity of combination of TPL and erastin, even in GSDME^low^ cancer cell

Our study showed that TPL treatment triggered GSDME-mediated pyroptotic cell death in cancer cells through repressing mitochondrial HK-II expression. TPL also suppressed NRF2/SLC7A11 axis and induced ROS accumulation in cancer cells, thus synergized with erastin to kill head and neck cancer cells, regardless of the expression status of GSDME (Fig. 9).

The anti-tumor activities of TPL have been previously linked to inducing mitochondrial apoptosis pathway and activation of caspases in cancers [20, 36–39]. In this study, we showed that TPL eliminated head and neck cancer cells mainly through GSDME-mediated pyroptotic cell death. We do observe that TPL treatment activated BAX-BAD mitochondrial pathway and active caspase 3 was responsible for cleavage of GSDME. It cannot be ruled out that pyroptosis induced by TPL in some cases have been mistakenly recorded as apoptosis in the past decades, because of activation of caspase 3 has been recognized as the hallmark of apoptosis for a long period. Pyroptosis exerts tumor suppressive role and links to establishment of anti-tumor immunity [40]. Induction of pyroptotic death in cancer cells by TPL may help to activate anti-tumor immunity and achieve long-term control of head and neck cancer.

GSDME is the major gasdermins family member that executes pyroptosis in epithelial cells [41]. Our data showed that GSDME is the major executor responsible for TPL induced pyroptosis in head and neck cancer cells. Until recently, it has been recognized that gasdermins pore forming proteins, not caspases, are determinant of cell death type [42]. In GSDME-expressing cells, active caspase 3 cleaves GSDME and liberates the pore-forming ability of N-terminus, resulting in cell swelling, membrane rupture, and pyroptotic cell death. However, activation of caspase 3 tends to induce apoptosis in GSDME-deficient cells [11]. Therefore, a possible explanation is that TPL may induce pyroptosis in GSDME^high^ cells, but induce apoptosis in GSDME-deficient cells. Although GSDMB is highly expressed in HK1 and FaDu cells, it seems that GSDMB did not contribute to TPL induced pyroptosis, because C666–1 cell, which has high level of GSDMB but without GSDME expression, was refractory to TPL induced pyroptosis. Unlike other gasdermins family proteins, the N-terminus of GSDMB by itself would not induce pyroptosis [43].

A unique characteristic of tumor cells is that they have a much higher demand for glucose than normal cells partially owing to the Warburg effect [44, 45]. HK-II binds to the out membrane of mitochondria via the Voltage-Dependent Anion Channel 1 (VDAC1) [46–48]. Mitochondrial HK-II is not only required for high glycolytic rate in cancer cell, but also plays an essential role in cell survival [49, 50]. Disassociation of HK-II from mitochondria elicits cell death [51, 52]. TPL consistently suppressed HK-II expression and glycolytic rates in HK1, FaDu and C666–1 cells, but failed to induce pyroptosis in GSDME-deficient C666–1 cell, suggesting that inhibition of HK-II activity is not sufficient to initiate pyroptosis in the absence of GSDME. Though its role in inhibition of mitochondrial BAX/BAD pathway is well known, our finding is the first to clarify the critical role of mitochondrial HK-II in preventing pyroptosis in cancer cells. HK-II is a transcriptional target of c-myc [53]. Our data showed that HK-II is transcriptionally regulated by c-myc. A pilot study indicated that TPL accelerated proteasomal degradation of c-myc protein in hepatocellular carcinoma [54]. Our data showed that TPL treatment not only shortened the half-life of c-myc protein, but also repressed the transcription of c-myc. Accelerated degradation of c-myc preceded transcriptional repression upon TPL treatment. Hyperactive transcription of c-myc is driven by super-enhancer in human cancers [55–57]. A recent study demonstrated that TPL disrupts super-enhancers in pancreatic cancer cells [58]. The levels of c-myc protein were decreased to undetectable level after TPL treatment for 24 h. This powerful inhibitory effect is a comprehensive result from simultaneously repression protein stability and transcription of c-myc by TPL.

GSDME is frequently silenced in human cancers due to hyper-methylation of promoter DNA [59], making cancer cells lack of GSDME are resistant to pyroptosis. It remains a challenge to kill cancers within dysfunction of cell death machinery. In addition to repressing c-myc/HK-II, TPL suppressed NRF2/SLC7A11 axis and led to accumulation of ROS in head and neck cancer cells. Though GSDME-deficient C666–1 cell was resistant to TPL induced pyroptosis, suppressing NRF2/SLC7A11 axis by TPL potentiated the cytotoxic effect of erastin in C666–1 cell, along with HK1 and FaDu cells. Xenograft tumor formation assays also revealed that combination TPL with erastin exhibited synthetic lethality on tumor cell. When this manuscript was under preparation, a group reported that TPL serves as NRF2 inhibitor and exhibits selective cytotoxicity in IDH1-mutated glioma cells [60]. Thus, our finding along with others strongly suggest that TPL provide a potent strategy for malignant therapy.

## Conclusions

Our study provided a novel paradigm of anti-tumor activity of TPL in head and neck cancers. Furthermore, this study highlighted a previously unrecognized function of mitochondrial HK-II in suppressing pyroptotic cell death in human cancers.

## Supplementary Information

**Additional file 1: Supplementary Table S1**. RT-PCR primers used in this study.

**Additional file 2: Supplementary Table S2**. Antibodies information used in this study.

**Additional file 3: Supplementary Table S3**. siRNA and shRNA used in this study.

**Additional file 4: Figure S1.** TPL transcriptionally and post-translationally suppresses c-myc expression. A, the mRNA levels of c-myc in head and neck cancer cells treated with TPL (50 nM) for 24 h were measured by qPCR. B, the protein levels of c-myc in head and neck cancer cells treated with TPL (50 nM) for 24 h were measured by western blot. C, immunofluorescence assays showed the powerful inhibitory effect of TPL (50 nM for 24 h) on c-myc protein. D, pulse-chase assay showed TPL (50 nM) shortened the half-life of translated c-myc protein. E, qPCR assays showed the mRNA level of c-myc started to decrease at 4 h later after TPL (50 nM) treatment.

**Additional file 5: Figure S2.** c-myc positively regulates HK-II in HK1 and FaDu cells. The mRNA levels of HK-II in c-myc silenced or overexpressed cells were determined by qPCR.

**Additional file 6: Supplementary materials S1.**

## Data Availability

The datasets used and/or analyzed during the current study are available from the corresponding author on reasonable request.

## Abbreviations

TPL: Triptolide
NPC: Nasopharyngeal carcinoma
EBV: Epstein-Barr virus
TWHf: *Tripterygium wilfordii Hook f*
ROS: Reactive oxygen species
DMSO: Dimethyl sulfoxide
PBS: Phosphate buffered saline
ATP: Adenosine triphosphate
IHC: Immunohistochemistry
HK- II: Hexokinase II
