# Correction to: Natural product triptolide induces GSDME-mediated pyroptosis in head and neck cancer through suppressing mitochondrial hexokinase-ΙΙ

**DOI:** 10.1186/s13046-021-02100-8

**Published:** 2021-09-22

**Authors:** Jing Cai, Mei Yi, Yixin Tan, Xiaoling Li, Guiyuan Li, Zhaoyang Zeng, Wei Xiong, Bo Xiang

**Affiliations:** 1 Hunan Key Laboratory of Cancer Metabolism, Hunan Cancer Hospital and the Affiliated Cancer Hospital of Xiangya School of Medicine, Central South University, Tongzipo Road, Changsha, 410013 Hunan China; 2 Hunan Key Laboratory of Nonresolving Inflammation and Cancer, The Third Xiangya Hospital, Central South University, Changsha, 410013 Hunan China; 3 The Key Laboratory of Carcinogenesis of the Chinese Ministry of Health, Xiangya Hospital, Central South University, Changsha, 410008 Hunan China; 4 The Key Laboratory of Carcinogenesis and Cancer Invasion of the Chinese Ministry of Education, Cancer Research Institute and School of Basic Medical Sciences, Central South University, Changsha, 410078 Hunan China; 5 Department of Dermatology, Xiangya Hospital, Central South University, Changsha, 410008 Hunan China; 6 Department of Dermatology, Hunan Key Laboratory of Medical Epigenetics, Second Xiangya Hospital, The Central South University, Changsha, 410011 Hunan China

**Correction to: J Exp Clin Cancer Res 40, 190 (2021)**

**https://doi.org/10.1186/s13046-021-01995-7**

Following publication of the original article [1], the authors identified some minor error in the Affiliation #1 details. The correct details are as follows:

^1^ Hunan Key Laboratory of Cancer Metabolism, ​Hunan Cancer Hospital and the Affiliated Cancer Hospital of Xiangya School of Medicine, Central South University, Tongzipo Road, Changsha, 410013, Hunan, China.

The correction does not have any effect on the results or conclusions of the paper. The original article has been corrected.
