# Supplementary material for: Natural product triptolide induces GSDME-mediated pyroptosis in head and neck cancer through suppressing mitochondrial hexokinase-ΙΙ
Source: J Exp Clin Cancer Res. 2021 Jun 9;40:190. doi: 10.1186/s13046-021-01995-7 (PMC8188724; doi:10.1186/s13046-021-01995-7)
Supplement: Supplementary file 1 — Additional file 1: Supplementary Table S1. RT-PCR primers used in this study. [file 13046_2021_1995_MOESM1_ESM.doc]

| Supplementary Table S1: RT-PCR primers used in this study | | |
| --- | --- | --- |
| Gene | Forward Primer(5'-3') | Reverse Primer(5'-3') |
| GSDMA | CCCGCTCCCAGAGACAATG | CGATGAGGCTGTCAAGTGGT |
| GSDMB | TCTCAGGGCCATCTCAGCTA | GCACCATCCTTCTCTTCTGGA |
| GSDMC | TCATTTGGATGGCCCTGGTG | CCAGGATGCTCCTTACCAGC |
| GSDMD | TCTGCCCTCAACACTTCTGG | TGCAGCCACAAATAACTCAGC |
| GSDME | CACACTGTGCCACTTGCTTC | GTCAGCTGAGGCAAACAAGC |
| PJVK | TGAAGCTTGAGACCCCGGAT | CAAAACGTCATCAACCAGCGT |
| NRF2 | AGGTTGCCCACATTCCCAAA | ACGTAGCCGAAGAAACCTCA |
| SLC7A11 | TGTGTGGGGTCCTGTCACTA | CAGTAGCTGCAGGGCGTATT |
| Bax | AAACTGGTGCTCAAGGCCC | AAAGTAGGAGAGGAGGCCGT |
| Bad | CTTGTCCTCACAGCCCAGAG | CCCCAGCGCCTCCATGA |
| caspase 3 | CTCTGGTTTTCGGTGGGTGT | CTTCCATGTATGATCTTTGGTTCC |
| c-Myc | GTAGTGGAAAACCAGCAGCC | AGAAATACGGCTGCACCGAG |
| HK-II | CATCCAGAGGAGAGGGGACT | TCATCGCCTTCCACCATGTC |
| GAPDH | AACGGATTTGGTCGTATTGG | TTGATTTTGGAGGGATCTCG |
