# Supplementary material for: Natural product triptolide induces GSDME-mediated pyroptosis in head and neck cancer through suppressing mitochondrial hexokinase-ΙΙ
Source: J Exp Clin Cancer Res. 2021 Jun 9;40:190. doi: 10.1186/s13046-021-01995-7 (PMC8188724; doi:10.1186/s13046-021-01995-7)
Supplement: Supplementary file 2 — Additional file 2: Supplementary Table S2. Antibodies information used in this study. [file 13046_2021_1995_MOESM2_ESM.doc]

| Supplementary Table S2. Antibodies information used in this study | | |
| --- | --- | --- |
| Antibodies | SOURCE | IDENTIFIER |
| anti-GSDME | Abcam | ab215191 |
| Caspase 3 | Cell Signaling Technology | 9665P |
| Cleaved Caspase 3 | Cell Signaling Technology | 9664P |
| cytochrome c | Santa Cruz | sc-13156 |
| anti-VDAC1 | Santa Cruz | sc-390996 |
| anti-NRF2 | Abclonal | WH148454 |
| Ki67 | ZSGB-BIO | ZA-0502 |
| polyclonal anti-Bax | Abclonal | A12009 |
| anti-Bad | Abclonal | A10753 |
| anti-Bak1 | Abclonal | A10754 |
| anti-c-Myc | Abcam | ab32072 |
| anti-HXK II | Santa Cruz | sc-374091 |
| anti-SLC7A11 | Abclonal | A13685 |
| PARP | Cell Signaling Technology | 9542 |
| Cleaved PARP | Cell Signaling Technology | 5625 |
| anti-GAPDH | BBI Life Sciences | D190090-0100 |
