# Supplementary material for: Natural product triptolide induces GSDME-mediated pyroptosis in head and neck cancer through suppressing mitochondrial hexokinase-ΙΙ
Source: J Exp Clin Cancer Res. 2021 Jun 9;40:190. doi: 10.1186/s13046-021-01995-7 (PMC8188724; doi:10.1186/s13046-021-01995-7)
Supplement: Supplementary file 3 — Additional file 3: Supplementary Table S3. siRNA and shRNA used in this study. [file 13046_2021_1995_MOESM3_ESM.doc]

| Supplementary Table S3: siRNA and shRNA used in this study | |
| --- | --- |
| siRNA | Sense(5'-3') |
| siBax#1 | GCTCTGAGCAGATCATGAA |
| siBax#2 | GCTTCAGGGTTTCATCCAG |
| sic-Myc#1 | AGTTGAAACACAAACTTGAAC |
| sic-Myc#2 | CATCTGACCTGGCATCTAATT |
| siCaspase3#1 | GGAACCAAAGATCATACAT |
| siCaspase3#2 | GCAGCAAACCTCAGGGAAA |
| siBad#1 | CCAGATCCCAGAGTTTGAG |
| siBad#2 | GGAGGATGAGTGACGAGTT |
| Scrambled | UUCUCCGAACGUGUCACGUTT |
| shGSDME#1 | Forward (5'-3'): CCGGTGATGATGGAGTATCTGATCTTCTCGAGAAGATCAGATACTCCATCATCTTTTTG  Reverse (5'-3'):  AATTCAAAAAGATGATGGAGTATCTGATCTTCTCGAGAAGATCAGATACTCCATCATCA |
| shGSDME#2 | Forward (5'-3'):  CCGGTCCTGATTGCAGTATCAAATCTCGAGATTTGATACTGCAATCAGGTTTTTG  Reverse (5'-3'):  AATTCAAAAACCTGATTGCAGTATCAAATCTCGAGATTTGATACTGCAATCAGGA |
| shHK-II#1 | Forward (5'-3'):  CCGGTCAAAGACATCTCAGACATTGCTCGAGCAATGTCTGAGATGTCTTTGTTTTTG  Reverse (5'-3'):  AATTCAAAAACAAAGACATCTCAGACATTGCTCGAGCAATGTCTGAGATGTCTTTGA |
| shHK-II#2 | Forward (5'-3'):  CCGGTGGAGCTCAACCATGACCAACTCGAGTTGGTCATGGTTGAGCTCCTTTTTG  Reverse (5'-3'):  AATTCAAAAAGGAGCTCAACCATGACCAACTCGAGTTGGTCATGGTTGAGCTCCA |
