# Supplementary figures and images for: Natural product triptolide induces GSDME-mediated pyroptosis in head and neck cancer through suppressing mitochondrial hexokinase-ΙΙ
Source: J Exp Clin Cancer Res. 2021 Jun 9;40:190. doi: 10.1186/s13046-021-01995-7 (PMC8188724; doi:10.1186/s13046-021-01995-7)

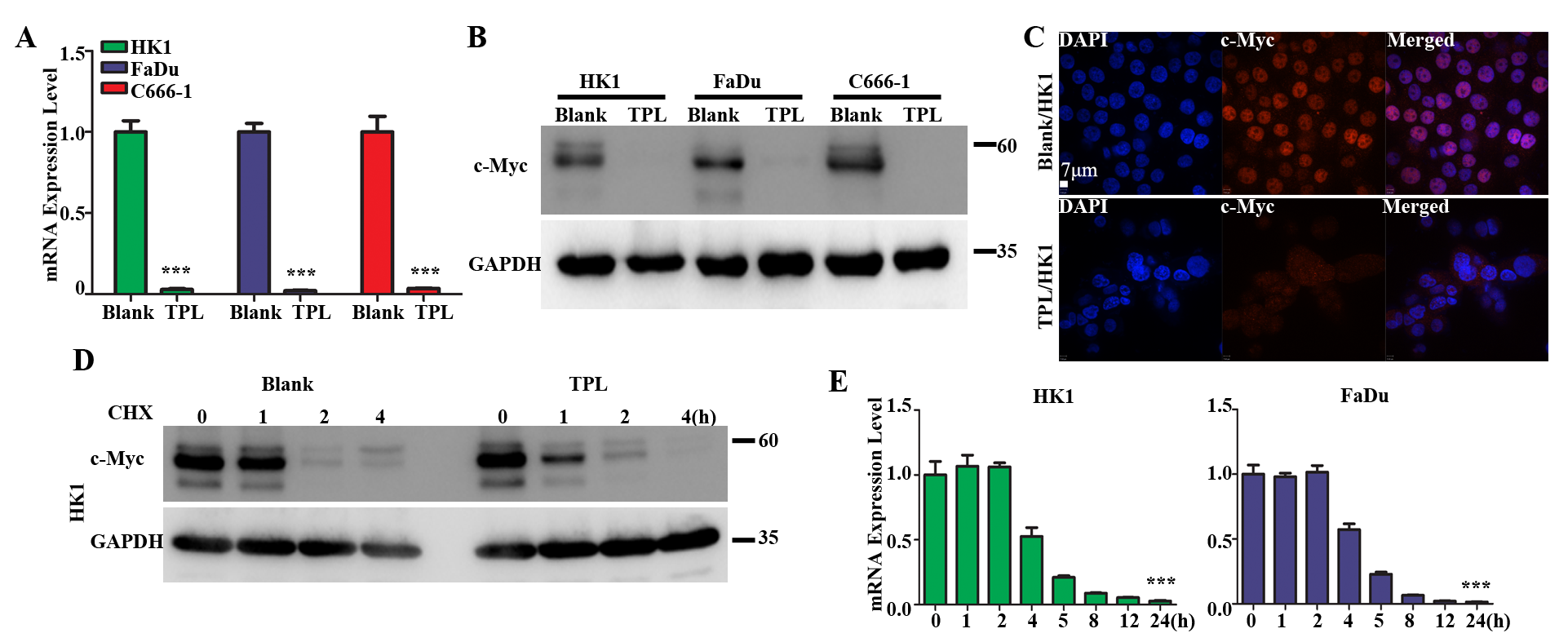

Supplement: Supplementary file 4 — Additional file 4: Figure S1. TPL transcriptionally and post-translationally suppresses c-myc expression. A, the mRNA levels of c-myc in head and neck cancer cells treated with TPL (50 nM) for 24 h were measured by qPCR. B, the protein levels of c-myc in head and neck cancer cells treated with TPL (50 nM) for 24 h were measured by western blot. C, immunofluorescence assays showed the powerful inhibitory effect of TPL (50 nM for 24 h) on c-myc protein. D, pulse-chase assay showed TPL (50 nM) shortened the half-life of translated c-myc protein. E, qPCR assays showed the mRNA level of c-myc started to decrease at 4 h later after TPL (50 nM) treatment. [file 13046_2021_1995_MOESM4_ESM.tif]

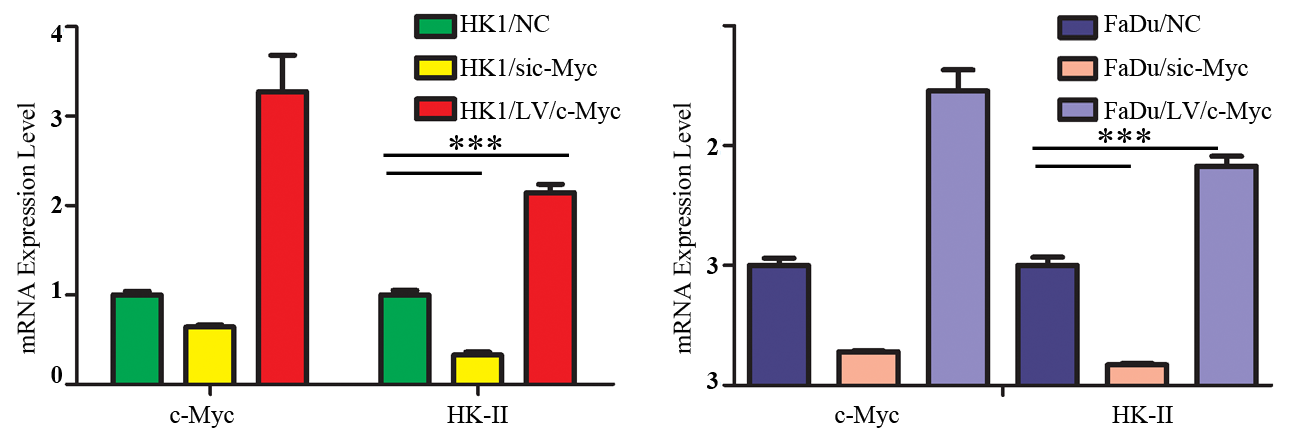

Supplement: Supplementary file 5 — Additional file 5: Figure S2. c-myc positively regulates HK-II in HK1 and FaDu cells. The mRNA levels of HK-II in c-myc silenced or overexpressed cells were determined by qPCR. [file 13046_2021_1995_MOESM5_ESM.tif]
